# Characterization and Expression Profiling of Neuropeptides and G-Protein-Coupled Receptors (GPCRs) for Neuropeptides in the Asian Citrus Psyllid, *Diaphorina citri* (Hemiptera: Psyllidae)

**DOI:** 10.3390/ijms19123912

**Published:** 2018-12-06

**Authors:** Zhengbing Wang, Wenwu Zhou, Muhammad Salman Hameed, Jiali Liu, Xinnian Zeng

**Affiliations:** 1 Guangdong Engineering Research Center for Insect Behavior Regulation, College of Agriculture, South China Agricultural University, Guangzhou 510642, China; wangzi191@yahoo.com (Z.W.); Shirley4462@scau.edu.cn (J.L.); 2 State Key Laboratory of Rice Biology; Institute of Insect Sciences, Zhejiang University, Hangzhou 310058, China; wenwuzhou@zju.edu.cn; 3 Department of Plant Protection, Faculty of Agricultural Sciences, Ghazi University, Dera Ghazi Khan 32200, Pakistan; msuleman2941@yahoo.com

**Keywords:** *Diaphorina citri*, expression profiling, G-protein-coupled receptors, transcriptome, neuropeptides, phylogenetic tree

## Abstract

Neuropeptides are endogenous active substances that widely exist in multicellular biological nerve tissue and participate in the function of the nervous system, and most of them act on neuropeptide receptors. In insects, neuropeptides and their receptors play important roles in controlling a multitude of physiological processes. In this project, we sequenced the transcriptome from twelve tissues of the Asian citrus psyllid, *Diaphorina citri* Kuwayama. A total of 40 candidate neuropeptide genes and 42 neuropeptide receptor genes were identified. Among the neuropeptide receptor genes, 35 of them belong to the A-family (or rhodopsin-like), four of them belong to the B-family (or secretin-like), and three of them are leucine-rich repeat-containing G-protein-coupled receptors. The expression profile of the 82 genes across developmental stages was determined by qRT-PCR. Our study provides the first investigation on the genes of neuropeptides and their receptors in *D. citri*, which may play key roles in regulating the physiology and behaviors of *D. citri*.

## 1. Introduction

The central nervous system (CNS) and its neuropeptide messengers rank the highest in the entity level regulating endogenous biochemical control function [1]. Neuropeptides are a diverse set of signaling molecules in multicellular organisms. In insects, neuropeptides play a significant role in the regulation of fundamental events such as development, reproduction, feeding, courtship, olfaction, circadian rhythm, and many other processes [2,3]. Neuropeptides are processed from their larger, inactive precursors by enzymes [4], up activation, and then work on target cells by binding to the specific signal-transducing membrane receptors [5]. Most of these receptors are subordinate to the G-protein-coupled receptors (GPCRs), and the GPCRs have a similar topographical structure with seven transmembrane domains which are highly conservative through evolution and constitute the largest superfamily of cell surface proteins [6]. In vivo studies showed that neuropeptides and their receptors appear to have key roles in the regulation of physiology and behavior in insects; injection of kinin caused a significant reduction in weight gain and an increased mortality in *Heliothis virescens* [7], injection of tachykinin (TK) resulted in diminished olfactory responses in electroantennograms of *Periplaneta americana* [8], reduced levels of the receptor of TK in *Drosophila melanogaster* olfactory receptor neurons (ORNs) showed increased attraction to two food-related odors [9]. Neuropeptides and their receptors regulate the fundamental events in insect life cycle, hence, they were proposed as potential insecticides or targets to replace or complement neurotoxic compounds [10]. Owing to the sequence specificity of these molecules and the specific receptor, neuropeptide-based compounds would be species‒specific, and safe for non-target species. Moreover, resistance is not easy to develop, since a potential mutation in a neuropeptide precursor or receptor gene is typically detrimental [11].

The Asian citrus psyllid, *Diaphorina citri* Kuwayama (Hemiptera: Psyllidae), is one of the most important insect pests of citrus worldwide. It is the principal vector of *Candidatus* Liberibacter asiaticus (CLas), a phloem-inhabiting bacterium, which correlates with Huanglongbing (HLB) [12]. Huanglongbing, also known as citrus greening disease, poses the most destructive threat to citrus production worldwide with no known cure [13,14,15]. At present, the main ways to prevent and restrain the spread of the disease are employing chemical insecticides to control the vector; however, the application of conventional insecticides, including organophosphates, pyrethroids, and neonicotinoids, has led to the evolution of resistance to pesticides [16,17,18]. The primary means of blocking the transmission of HLB is to control *D. citri*, the vector of the pathogen; however, the development of insecticide resistance in *D. citri* also poses increased challenges for the persistent effectiveness of chemical control strategies. Furthermore, the more use of pesticides can also lead to problems with “resurgence” and “residue”. Pesticides with safe and novel ways of action are needed to alleviate the problems of pesticide resistance and environmental pollution. The primary task of developing neuroendocrine-based insecticides is to identify the structure and function of neuropeptides and the receptors involved in survival, development, and/or reproduction [11].

Previous studies have identified the neuropeptides or neuropeptide receptors in *D. melanogaster* [3], *Bombyx mori* [19], *Chilo suppressalis* [20], *Tribolium castaneum* [21], *Apis mellifera* [22], *Nilaparvata lugens* [23], and *Rhodnius prolixus* [24]. However, the neuropeptides and their GPCRs in *D. citri* are poorly characterized. For these reasons, in this study, to identify gene-encoding neuropeptides and the receptors of neuropeptides in *D. citri*, we analyzed the transcriptomes in twelve tissues of this pest. The expression profiles of these neuropeptides and the receptors were validated by quantitative real time-PCR (qRT-PCR). This is the first study on the neuropeptides and neuropeptide receptors of *D. citri*, and these results provide valuable information to develop an integrated pest management program for the Asian citrus psyllid.

## 2. Results

### 2.1. Sequencing and Unigene Assembly

**Figure 1:**
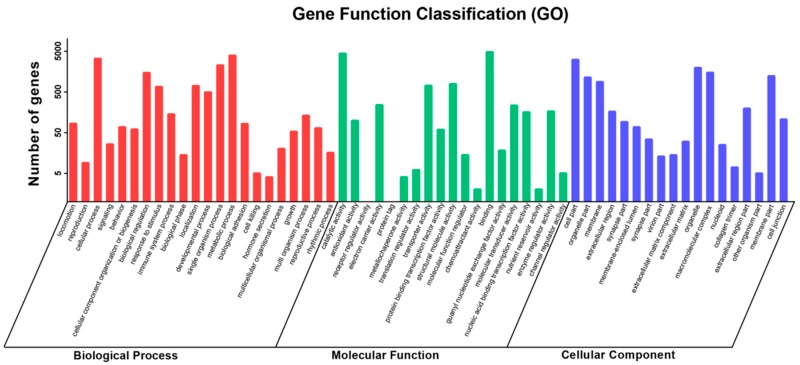
Gene Ontology (GO) classifications of unigenes. The *y*-axis represents the number of the unigenes, the *x*-axis shows three categories and their subcategories.

A total of 143.37 Gb of raw data was acquired, and after removing low-quality, adaptor, and contaminated sequence reads, yielded 137.22 Gb of clean reads. These clean reads were assembled into 38,161,895 contigs (N50 = 48) of an average length of 50 bp by Trinity. Then we obtained 297,614 unigenes larger than 200 bp (N50 = 731) from non-redundant putative contigs, with a mean length of 575 bp. For annotations, the unigenes were searched against Nr (NCBI non-redundant protein sequences, http://www.ncbi.nlm.nih.gov/), Swiss-Prot (a manually annotated and reviewed protein sequence database, http://www.expasy.ch/sprot/), KEGG (Kyoto Encyclopedia of Genes and Genomes, http://www.genome.jp/kegg/), COG (Clusters of Orthologous Groups of proteins, http://www.ncbi.nlm.nih.gov/COG/), and GO (Gene Ontology, http://www.geneontology.org/) databases using BLASTx (search protein databases using a translated nucleotide query) with a cut-off E value of 10^−5^. As a result, 60,401 (20.29%) were annotated using the NCBI-Nr database; 34,380 (11.55%) by Swiss-Prot; 11,837 (3.98%) by KEGG; 30,808 (10.35%) by COG; 8691 (2.92%) by GO, which covered 60,999 (20.49%) of the total unigenes. Of the 297,614 unigenes, 8691 (2.92%) matched at least one GO term (Figure 1). Among these unigenes, 13,536, 11,569, and 10,489 transcripts were assigned to biological processes, molecular function, and cellular component, respectively. Cellular process and metabolic processes represented the most abundant GO terms in the biological process category, binding and catalytic activity were most represented in molecular function, and the most unigenes that accorded with the cellular component category were involved in cell parts and organelles (Figure 1). The raw data of the transcriptomic were submitted to the NCBI Short Read Archive (SRA) database as BioProject Accession Number SRP139008 (https://www.ncbi.nlm.nih.gov/sra/SRP139008).

### 2.2. Neuropeptide and Peptide Hormone Genes

Based on Nr-annotation and homology searches, 40 genes encoding neuropeptides and neurohormones were identified in *D. citri* (Table 1), including the neuropeptides involved in physiology and behavior, such as allatotropin (AT), crustacean cardioactive peptide (CCAP), CCHamide (CCH), ecdysis triggering hormone (ETH), eclosion hormone (EH), SIFamide (SIF), pigment-dispersing factor (PDF), tachykinin (TK) and natalisin (NTL). Elevenin-like peptide (ELP) is a molluscan neuropeptide and was first identified in *Aplysia californica*, and later ELP was found in many insect species with the exception of *D. melanogaster* and *B. mori*, in *D. citri*, the gene coding for ELP was found. Prothoracicotropic hormone (PTTH) has been proposed to regulate development in insects, and it has been identified in *D. citri*, however, previously, in Hemipteran, PTTH was only identified in *N. lugens*. The adipokinetic hormone (AKH)/corazonin-related peptide (ACP), bursicon alpha subunit (Burα), FMRFamide (FMRF), glycoprotein hormone alpha 2 (GPA2), IMFamide (IMF), neuropeptide F (NPF), sulfakinin (SK), and trissin (TR) were not found in the transcriptome databases of *D. citri* (Supplementary Table S1).

### 2.3. G-Protein-Coupled Receptors (GPCRs) for Neuropeptides

**Figure 2:**
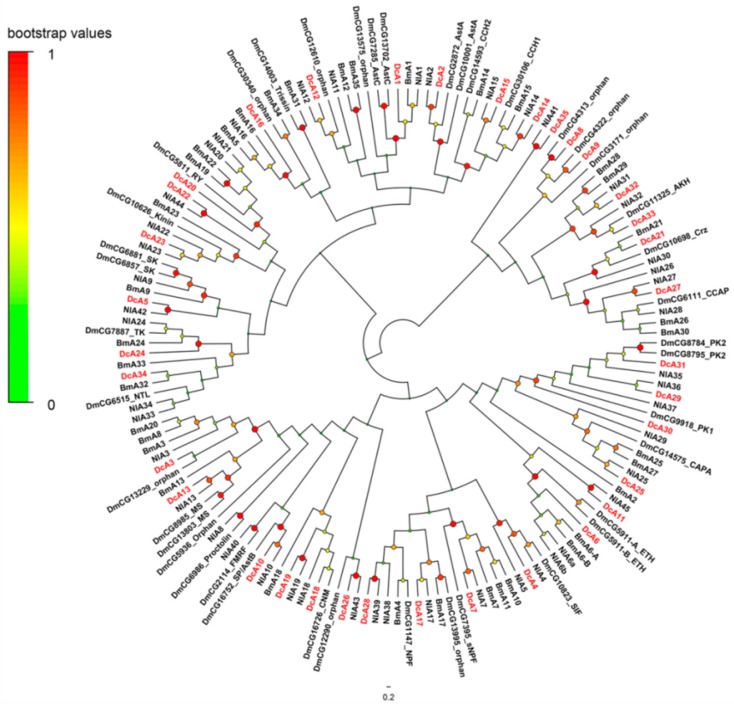
Phylogenetic tree of the A-family neuropeptide GPCRs. The neighbor-joining trees were constructed using MEGA 6 software with 1000-fold bootstrap repetitions. The A-family neuropeptide GPCRs of *D. citri* are shown in red text. Dc, *D. citri*; Dm, *D. melanogaster*; Nl, *N. lugens*.

**Figure 3:**
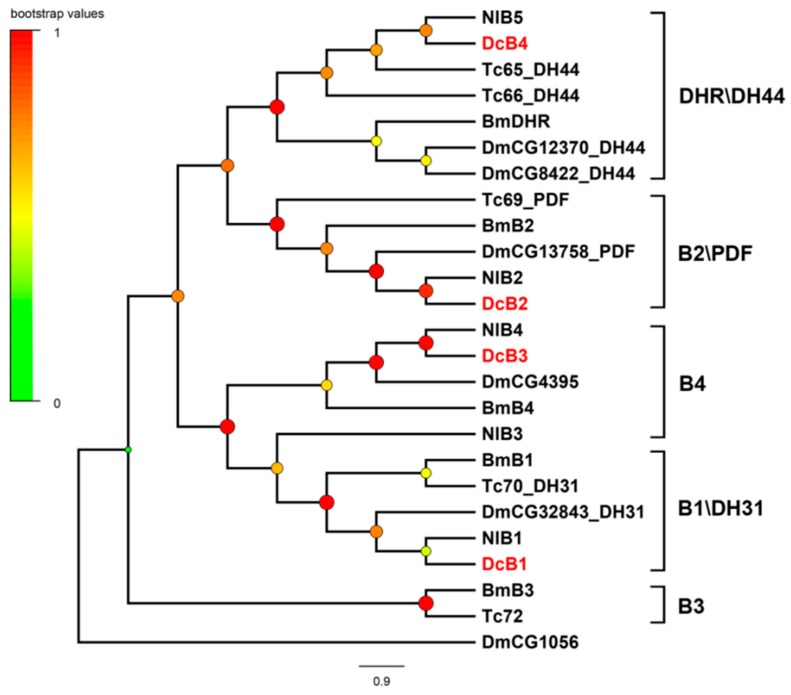
Phylogenetic tree of the B-family neuropeptide GPCRs. The neighbor-joining trees were constructed using MEGA 6 software with 1000-fold bootstrap repetitions. The B-family neuropeptide GPCRs of *D. citri* are shown in red text. Dc, *D. citri*; Dm, *D. melanogaster*; Nl, *N. lugens*; Bm, *B. mori*; Tc, *T. castaneum*.

**Figure 4:**
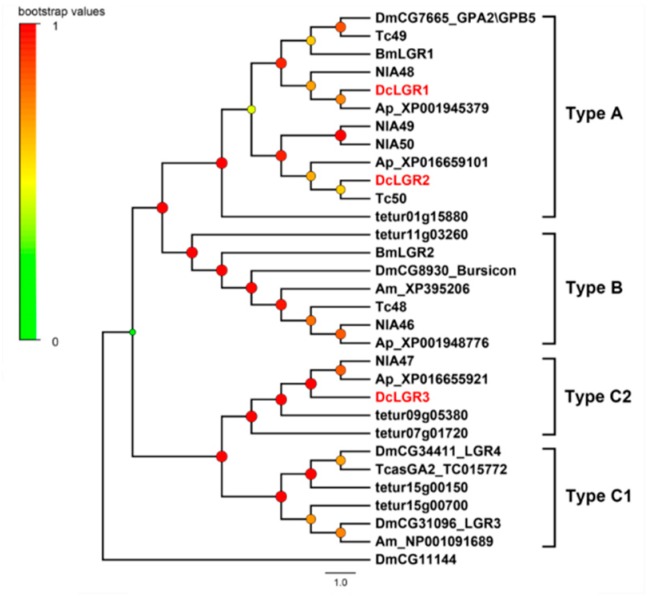
Phylogenetic tree of leucine-rich repeat-containing GPCRs (LGRs). The neighbor-joining trees were constructed using MEGA 6 software with 1000-fold bootstrap repetitions. The LGRs of *D. citri* are shown in red text. Dc, *D. citri*; Dm, *D. melanogaster*; Nl, *N. lugens*; Bm, *B. mori*; Tc, *T. castaneum*. Ap, *Acyrthosiphon pisum*; Am, *A. mellifera*; tetur, *Tetranychus urticae*.

A total of 42 putative neuropeptide GPCRs genes were identified in the transcriptomes of *D. citri* based on homology analysis (Table 2). Of these receptors, 35 GPCRs belong to the A family, four belong to B family, and three belong to leucine-rich repeat-containing GPCRs (LGRs). In order to assign putative functions of these GPCRs, we compared them with those of *D. melanogaster*, *N. lugens*, and other arthropods. The results are presented as a neighbor-joining tree in Figure 2, Figure 3 and Figure 4.

#### 2.3.1. A-Family GPCRs

The A-family GPCRs are also known as the rhodopsin family. Thirty-five A-family neuropeptide GPCRs were identified in *D. citri*. In the phylogenetic tree of A-family GPCRs (Figure 2), DcA5, DcA20, DcA22-24, and DcA34 were clustered in a clade with DmCG5811 (RYamide receptor, RYR), DmCG10626 (Kinin receptor, KinR), DmCG6857 (Sulfakinin-like receptor, SKR), DmCG6881 (Sulfakinin-like receptor, SKR), CG7887 (Tachykinin receptor, TKR), DmCG6515 (Natalisin receptor, NTLR). DcA5, DcA23, DcA24, and DcA34 were identified as SKR, KinR, TKR, and NTLR respectively. DcA22 and DcA20 are two orthologs of DmRYaR. DcA7, DcA17, and DcA28 stay in the clade of NPF and short Neuropeptide F (sNPF) receptors, DcA28 is the receptor of NPF (NPFR), and DcA7 and DcA17 were identified as sNPF receptors (sNPFR). Besides that, there is another big clade locating in the phylogenetic tree, and it contains the Pyrokinin receptor (PKR), cardio acceleratory peptide (CAPAR), ecdysis-triggering hormone receptor (ETHR). DcA6 was predicted as ETHR, and DcA25 acts as a CAPAR, based on the Blastp analysis and phylogenetic analysis. DcA25 and DcA30 were identified as PK1R, DcA31 was identified as PK2R. The receptors of the GnRH-related peptides and CCAP were clustered in a clade, including DcA21, DcA27, DcA32, and DcA33. DcA21 located in the subtree of the corazonin receptor with BmA21, DcA27 was identified as CCAPR. DcA32 and DcA33 act as the receptor of AKH. DcA8, DcA9, and DcA35 are paralogs of CCAPR and AKHR; however, DcA8 and DcA9 are similar to two orphan receptors, DmCG4313 and DmCG4322. DcA35 is similar to NlA41, and NlA41 is an ortholog of the receptor for AVLP of *T. castaneum*, further based on blastp analysis, DcA35 has a high homology with AVLPR of *T. castaneum* (ABX00684, e-value 3e-112). As above, DcA35 was characterized as DcAVLPR. DcA11 is an ortholog of NlA45, and NlA45 is remarkably similar to the vertebrate thyrotropin-releasing hormone receptors (TRHRs). The receptors for CNMamide (CNM), FMRF, Myosuppressin (MS), sex peptide (SP), Proctolin (Pro), and 3 orphan receptors, DmCG12290, DmCG5986, and DmCG13229 were clustered in a clade. This clade included DcA3, DcA10, and DcA13. DcA10 was identified as the receptor of SP (SPR), and DcA13 acts as MS receptor (MSR). The predicted function of DcA3 was not identified. At last, DcA1, DcA2, DcA4, and DcA15 were obviously identified as receptors of AstC, AstA, SIF, and CCH, respectively.

#### 2.3.2. B-Family GPCRs

B-family GPCRs contain three subfamilies: subfamily B1, subfamily B2, and subfamily B3. Here the B-family neuropeptide GPCRs belong to the subfamily B1. Four B-family GPCRs associated with neuropeptide recognition were confirmed in *D. citri*. Each amino acid sequence of these candidate receptors contains the characteristic hormone receptor domain. In the phylogenetic tree of B-family GPCRs (Figure 3), four receptors were classified into four branches. DcB1 and DcB4 were identified as receptors of DH31 and DH44. DcB1 is the ortholog of DmCG32843, and DcB4 is the ortholog of CG12370 and DmCG8422. DcB2 was annotated as the receptor of PDF, which is involved in prolonging mating duration. DcB3 is an ortholog of DmCG4395, and it shows high similarity to the calcitonin-like diuretic hormone receptor 2 of *R. prolixus* (AHB86571, e-value 0.0).

#### 2.3.3. Leucine-Rich Repeat-Containing GPCRs (LGRs)

Leucine-rich repeat-containing GPCRs are a class of GPCRs containing leucine-rich repeats (LRRs) at the N-terminus. Based on the number of LRRs, the type-specific hinge region and the presence or absence of a low-density lipoprotein receptor-like cysteine-rich motif (LDLa), LGRs can be identified as three main types (type A, B, and C). Furthermore, according to the number of LDLa motifs, Type C LGRs can be divided into two subtypes: Type C1, which contain only one LDLa, and Type C2, which contain multiple LDLa motifs. Three LGRs were identified in *D. citri*, two belong to Type A and one belong to Type C2. Both DcLGR1 and DcLGR2 contain eight LRRs, and this is a typical feature of Type A. In the phylogenetic tree of leucine-rich repeat-containing GPCRs (Figure 4), DcLGR1 and DcLGR2 are homologous to DmCG7665 which is the receptor of glycoprotein hormones in *Drosophila*. DcLGR3 contains eight LDLa motifs and two LRRs, and it belongs to Type C2. DcLGR3 is an ortholog of NlA47 which is also known as NlGRL101.

### 2.4. Tissue-Specific Expression Profiles of the Neuropeptides and Neuropeptide Receptors

**Figure 5:**
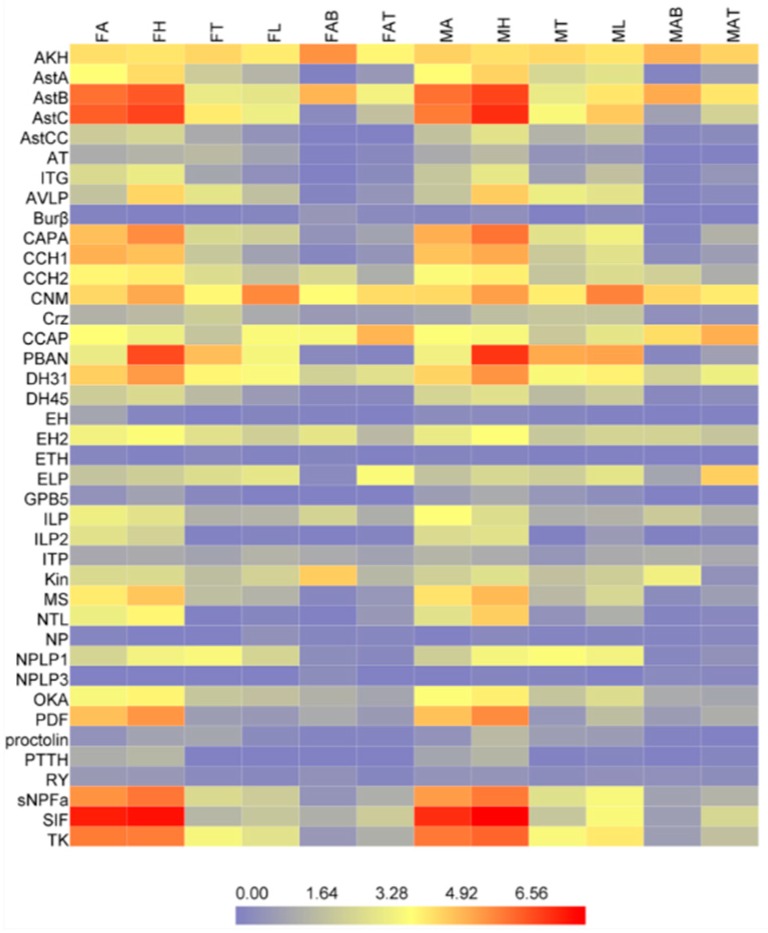
Expression profiles of neuropeptides in various tissues of *D. citri* based on FPKM values (fragments per kilobase per million reads). The mRNA levels, as represented by log2 (FPKM + 1) values, are shown in a heat map with colors ranging from blue (low expression) to red (high expression). MA, male antenna; MH, male head; MT, male thorax; ML, male leg; MAB, male abdomen; MAT, male abdomen terminal; FA, female antenna; FH, female head; FT, female thorax; FL, female leg; FAB, female abdomen; FAT, female abdomen terminal.

In order to understand the potential function of the neuropeptides and neuropeptide receptors in *D. citri*, the expressions of the neuropeptides and neuropeptide receptors were profiled for different tissues of *D. citri* based on the transcriptome data (Figure 5). AKH, DH31, SIF, AstB, CCH2, ILP, EH2, CNM, ELP, CCAP, and Kin are ubiquitous in the tissues of the female and male adult. AstA-C, TK, CAPA, ITG, CCH1-2, MS, sNPF, OKA, ILP1-2, SIF, NTL, EH2, AVLP, PBAN, NPLP1, and CNM show high expression levels in head, and AstA, CAPA, ITG, IPL2, NTL; PDF express in head and antenna; CNM also shows the highest expression level in legs of all tissues; and ELP and CCAP have a high expression level in abdomen terminal.

**Figure 6:**
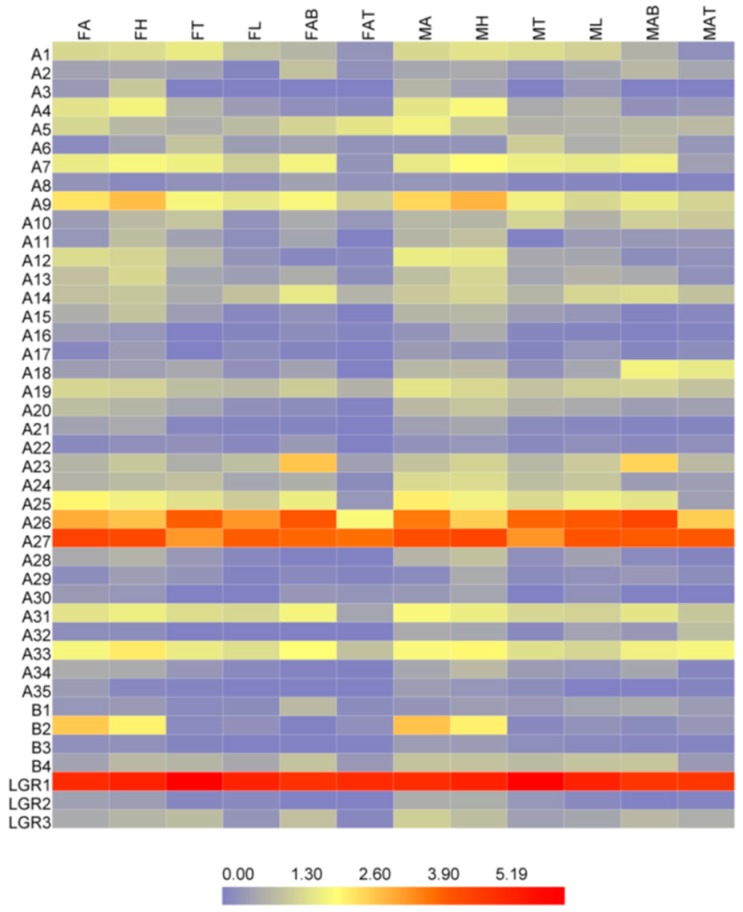
Expression profiles of neuropeptide receptors in various tissues of *D. citri* based on FPKM values. The mRNA levels, as represented by log2 (FPKM + 1) values, are shown in the heat map with colors ranging from blue (low expression) to red (high expression). MA, male antenna; MH, male head; MT, male thorax; ML, male leg; MAB, male abdomen; MAT, male abdomen terminal; FA, female antenna; FH, female head; FT, female thorax; FL, female leg; FAB, female abdomen; FAT, female abdomen terminal.

The overwhelming majority of the neuropeptide receptors appear to be lowly expressed in all tissues in addition to A4, A7, A9, A12, A18, A23, A26, A27, A31, A33, B2, LGR1 (Figure 6). LGR1 (glycoprotein hormones), A26, A27 (CCAP) have the highest expression levels of all the receptors and exist in all tissues of the female and male adult. A7 (sNPF), A9, A25 (CAPA), and A31 (PBAN) express broadly in the head, thorax, and abdomen except the abdomen terminal. A4 (SIF), A12, and B2 (PDF) express mainly in the head and antenna. A23 (kinin) show the specific expression in the abdomen of female and male. A18 (CNM) especially expresses in the abdomen and abdomen terminal of the male. A24 (TK) have higher expressions in the head and antenna of males than other tissues.

### 2.5. Developmental Stages Expression Analysis by RT-qPCR

**Figure 7:**
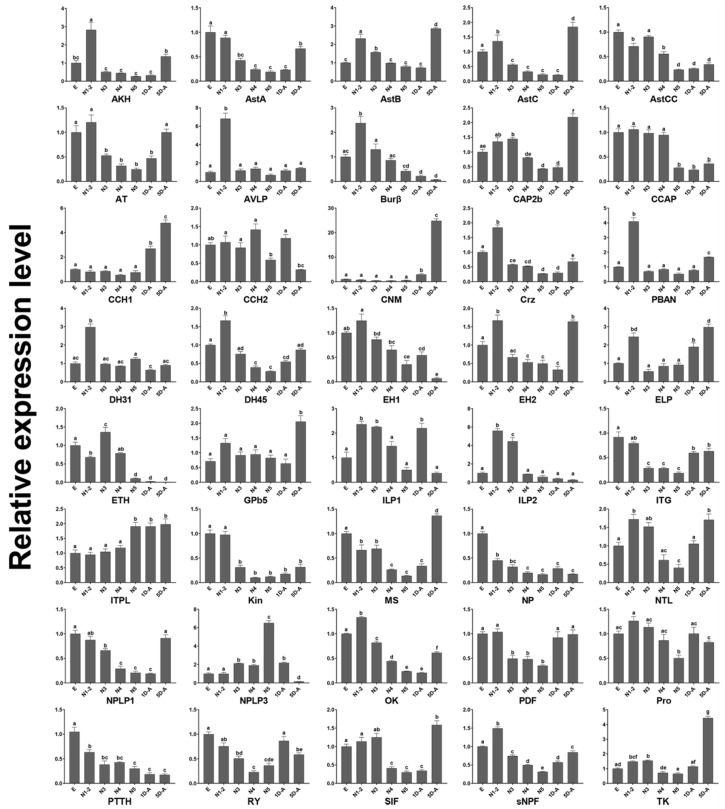
qRT-PCR results of neuropeptides throughout the *D. citri* life cycle. The *y*-axis represents the relative expression level and the *x*-axis the life cycle. The standard error is represented by the error bar and significant differences are represented by the different letters (*p* < 0.05). E, egg; N1-2, 1st- and 2nd-instar nymphs; N3, 3rd instar nymphs; N4, 4th instar nymphs; N5, 5th instar nymphs; 1D-A, the adults of one day after eclosion; 5D-A, five days after eclosion.

**Figure 8:**
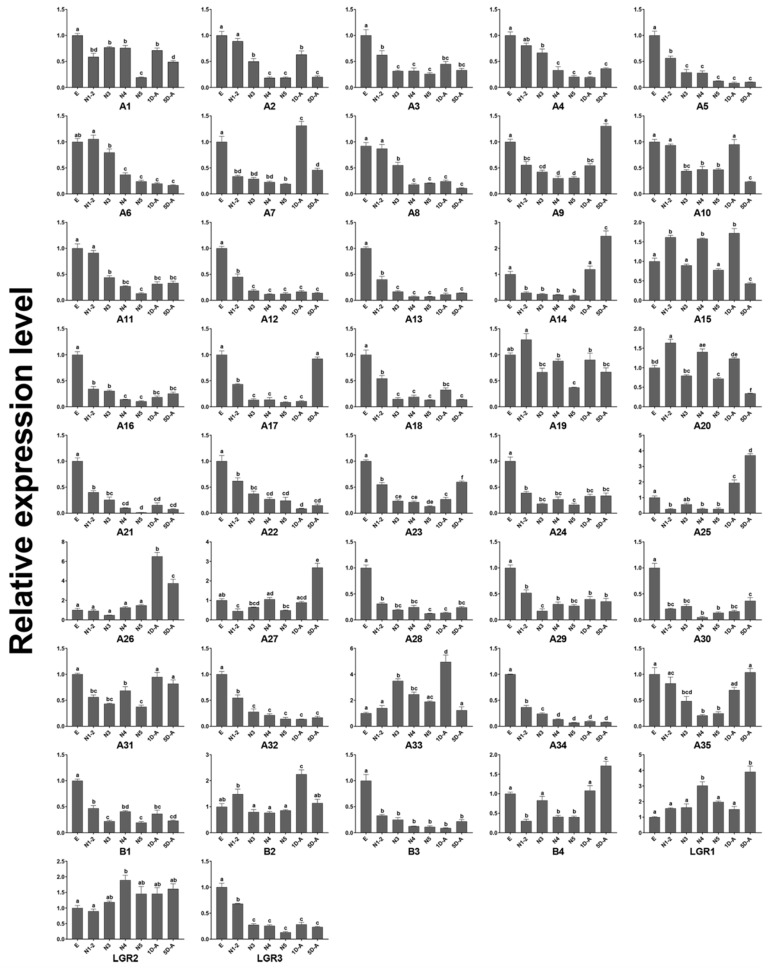
The gene expression level of neuropeptide receptors throughout the *D. citri* life cycle. The *y*-axis represents the relative expression level and the *x*-axis the life cycle. The standard error is represented by the error bar and significant differences are represented by the different letters (*p* < 0.05). E, egg; N1-2, 1st- and 2nd-instar nymphs; N3, 3rd instar nymphs; N4, 4th instar nymphs; N5, 5th instar nymphs; 1D-A, the adults of one day after eclosion; 5D-A, five days after eclosion.

Developmental stages expression profiles of the neuropeptides and neuropeptide receptors were confirmed by the RT-qPCR (Figure 7 and Figure 8). AKH, AVLP, Burβ, DH31, DH45, PBAN, and OK had the highest expressional level in the 1st and 2nd instar nymphs. AstB, AstC, CAP2b, CCH1, CNM, GPb5, MS, and TK showed the highest expression of the adults at five days after eclosion (sexual maturity). The expression level of Kin, NP, and PTTH decreased gradually from egg to sexual maturity. The neuropeptide receptors A3, A5, A12, A13, A16, A18, A21–24, A28–A30, A32, A34, B1, B3, and LGR3 were predominately expressed in egg, and A14, A25–27, and B4 mainly expressed in adults. A35 and two orphan receptors (A9 and A17) both expressed highly in egg and adult.

## 3. Discussion

Control of *D. citri* is the key component of integrated control for citrus Huanglongbing. However, effective control measures are not currently available. As potential pesticides and new targets emerge on account of continuing development of existing insecticide resistance [1,25], the primary task of developing neuroendocrine-based insecticides is to identify the structure and function of neuropeptides and receptors, especially those involved in survival, development, and/or reproduction [11]. However, the molecular basis of the behavior of *D. citri* is poorly understood. Besides, the lack of genomic information prevents us from understanding the regulatory network of the neuropeptides. Fortunately, transcriptome analysis has provided the methods to identify and characterize multiple genes in insects [23], and using RNA-seq a number of genes encoding neuropeptides and their receptors were identified from *D. citri*.

The present transcriptional sequences of *D. citri* appear to contain most of the genes for insect neuropeptides and GPCRs except for several genes. Several neuropeptides (e.g., Trissin (TR) and IMFamide (IMF) orthologs) were also not identified in other Hemipteran insects such as *A. pisum* [26], *N. lugens* [23], and *R. prolixus* [11]. In fact, IMF is a unique neuropeptide of Lepidoptera and has not been found in other insects [20]. Neuropeptide F (NPF) is identified in almost all insect species and plays roles in feeding, metabolism, reproduction, and stress responses [20], whereas the BLAST search of transcriptomic data failed to find NPF in *D. citri*. Similarly, we have identified an ortholog of Proctolin; however, the receptor was not found. This may be due to the incomplete analyses of transcriptome information. Indeed, the gene coding for natalisin (NTL) and CNMamide (CNM) were identified from *R. prolixus* by reinvestigation [11] contrary to the previous suggestion that NTL and CNM were absent [24].

### 3.1. Neuropeptides Involved in Ecdysis and Development

Ecdysis-triggering hormone (ETH), eclosion hormone (EH), and crustacean cardioactive peptide (CCAP) are main players of the peptidergic circuit controlling ecdysis in insects. The functions of these peptides have been reported in the *Drosophila* and other insects [27,28]. In insects, EH is expressed in CNS, ETH production in endocrine cells in the epitracheal gland (Inka cells), and the CCAP-expressing neurons are located in abdominal ganglia [27]. Both ETH and EH regulate the release of CCAP from central CCAP neurons which inhibits pre-ecdysis [2]. In *D. citri*, one ETH encoding gene was identified, and two different genes encoding EH were identified. According to the expression profiles, ETH and EH1 show high expression levels in the nymph stage, and we can presume that ETH and EH1 play a vital role in molting processes.

Juvenile hormone (JH) is an important hormone and regulates development and growth in insects. Traditionally JH production in the corpora allata has been considered to be controlled by the peptides Allatotropins (AT) [29] and Allatostatins (Ast) [2]. The first insect AT was isolated from head extracts of *Manduca sexta*. This peptide was shown to stimulate JH biosynthesis by the corpora allata (CA) of adult females [29]. Allatostatins are diverse peptides derived from three different genes in insects, which were designated as AstA, AstB, and AstC. These peptides act as the inhibitors of JH production in corpora allata (CA) and have antagonism to AT [30]. Feeding the Lepidopteran AstC led to reduced growth and fecundity and caused significant mortality in *A. pisum* and in *Myzus persicae* [31,32]. The function of AstC receptors has been characterized in *A. aegypti* and *T. castaneum*, and they express in the central nervous system and gut [33,34]. Besides that, AstCC is a Type-C Ast and shows strong similarities to AstC. AstCC also has the function to regulate the biosynthesis of JH [35]. In *D. citri*, from 3rd instar nymphs to sexual maturity, the expression of AstCC shows a decreasing trend, and AT shows a growth trend. This result suggests that AT and AstCC might be involved in the regulation of JH production in *D. citri*.

### 3.2. Neuropeptides Control of Metabolism

Insulin-like peptides (ILPs) widely exist in insects and the insulin-like signaling pathway is conserved across higher multicellular animals [36]. In *Drosophila*, ablation of the insulin-producing cells, or deactivation of the ILPR leads to a series of phenotypes including grow logy, increased starvation resistance, increased levels of circulating carbohydrates cycling, elevated lipid storage, and lifespan extension [2]. Adipokinetic hormone (AKH) is suggested to be similar to mammalian glucagon and acts antagonistically to insulin by activating glycogen phosphorylase and mobilizing carbohydrates [37]. When the AKH-producing neuroendocrine cells were ablated in *Drosophila*, the trehalose levels of larvae and starved adults decreased, and these adults without AKH-cells become hypoactive, suggesting that AKH is involved in maintaining normal levels of circulating carbohydrates [38]. Ectopic expression of AKH in the fat body resulted in both increased circulating trehalose and a decrease in stored lipids [2]. In *D. citri*, two genes encoding ILP1 and ILP2 and one gene encoding AKH were identified, and more research is needed to reveal the functions of these genes.

The insect kinins are multifunctional neuropeptides shown to modulate hindgut contractions [39,40], diuretic activity [41,42], digestive enzyme release [40,43], and inhibit weight gain in larvae Lepidoptera [43,44]. Feeding the analogs of kinin to the pea aphid demonstrated that three of the biostable analogs showed antifeedant activity [45]. In *D. citri*, we identified one kinin precursor and one kinin receptor (DcA23), the kinin and the kinin receptor both express in the abdomens (Figure 5 and Figure 6). On the present understanding of the expression profile of Kin and the receptor, we speculate that kinin and DcA23 may be involved in regulating the digestion process in *D. citri* and they represent a potential pesticide and target.

### 3.3. Reproductive-Related Neuropeptides

SIFamide (SIF) is strictly conserved and widespread in insects and modulates sexual behavior, and it was first found in 1995 in HPLC (high-performance liquid chromatography) fractions [46]. In *Drosophila*, the expression of SIF was restricted to only four neurons of the pars intercerebralis, when the SIF neurons was ablated, the lacking SIF-less males perform vigorous and indiscriminate courtship directed at either sex, while females appear sexually hyper-receptive. When the SIF gene was knocked down via RNAi, the decrease of SIF also led to a similar change of behavior [47]. SIFamides show a conserved sequence, X1-X2-RKPPFNGSIFamide, and the SIFs differ only in their N-terminal amino [46], in *D. citri*, SIF and the receptor of SIF (DcA4) were detected.

Neuropeptide pigment-dispersing factor (PDF) is involved in maintaining behavioral rhythms in *D. melanogaster* [48]. In addition, males usually prolong mating duration in the presence of other males to increase the chance of successful gene transfer, this effect in *D. melanogaster* requires both Neuropeptide F receptor 1 (NPFR1) and PDF expressing in four small ventrolateral neurons as well as the PDF receptor and expressing Neuropeptide F (NPF) in two dorsolateral neurons [49]. Apparently, PDF and NPF work together to regulate prolonged mating duration in *D. melanogaster*. In this study, PDF was identified in *D. citri*; however, NPF was not found.

Natalisin (NTL) is an arthropod-specific neuropeptide which was recently identified in three holometabolous insect species: *D. melanogaster*, *T. castaneum*, and *B. mori*, and was proven to be involved in regulating mating behavior in *D. melanogaster* and *T. castaneum* [50]. The latest research on NTL shows that NTL is involved in modulating the mating of the oriental fruit fly, *Bactrocera dorsalis* [51]. The NTL precursors generally contain multiple repeats sequences of F-X1-X2-X3-Ra at the C-terminus. In hemipteran, X1, is usually W, and X2 is P [52]. In *D. citri*, NTL precursors and the receptor of NTL (DcA34) was identified, according to the amino acid sequences of the NTL precursor, four mature peptides were predicted (Supplementary Figure S1). The conservatism of NTLs sequences implies the conservatism of the functions; NTL will be a worthwhile option in developing novel control methods against *D. citri*.

### 3.4. Neuropeptides in Olfaction

Tachykinin (TK) is a multifunctional peptide, and it has been identified in many vertebrate and invertebrate species. In all vertebrate and a few invertebrate the TKs share a common C-terminal sequence motif, F-X-G-L-Ra [53]. Tachykinin is important in odor-based searching behavior of fruit flies, and several olfactory neurons contain high TK levels [54]. In *Drosophila*, (3R,11Z,19Z)-3-acetoxy-11,19-octacosadien-1-ol (CH503) is a gustatory sex pheromone. CH503 is detected by gustatory neurons on the male foreleg, a cluster of 8–10 neurons within the subesophageal region which mediates the pheromone response through the release of the TKs [55]. In *D. citri*, we identified four mature peptides of TK; all mature peptides have conserved sequences at the C-terminus (Supplementary Figure S1). Tachykinin shows a high expression level in antenna and DcA24 shows high expression in male antenna and head in *D. citri*, but beyond that, TK also showed the highest expression in adults at five days after sexual maturity. Based on the expression profile, it is probable that TK and DcA24 are involved in the recognition of the odor by adults. Therefore, TK and the receptor can be considered as a potential behavioral regulatory pesticide or target.

CCH1 and CCH2 were initially identified in the tsetse fly *Glossina morsitans* [56]. In *Drosophila*, CCH1 and CCH2 played a role in appetite regulation by activating their receptors respectively [57,58]. The receptor of CCH1 was also an important factor governing starvation-induced olfactory modifications [59]. Here, both CCH1 and CCH2 were identified in *D. citri*, and their corresponding receptors were excavated, A14 (CCH1) and A15 (CCH2), respectively. In *D. citri*, CCH1 and A14 presented high expression in adulthood, besides that, CCH1 is highly expressed in antennae and head. According to the results, it was assumed that CCH1 and A14 may play an important role in regulating starvation-induced host recognition behavior at the adult stage of *D. citri*. In addition to TK and CCHs, AstA, sNPF, and SIF also show high expression level in antenna and head, and it is consistent with neuropeptides expressed in brain and the antennal lobe of other insects as in previous reports [47,60,61].

## 4. Materials and Methods

### 4.1. Insect Rearing and RNA Extraction

The insects were collected from the *Murraya exotica* in the campus of South China Agricultural University, Guangzhou, Guangdong Province, China in 2013. The laboratory population of *D. citri* was reared in a greenhouse (26 °C, 80% RH) and 14:10 h (light:dark) photoperiod. *D. citri* adults were transferred to cages (40 × 40 × 50 cm) which contained the saplings of *M. exotica* for oviposition and feeding 4 days, the plants with eggs were transferred to new cages. When the eggs hatched, each instar of *D. citri* was collected during the process of growth and tissues of insect dissected from newly emerged adults (3 days old). Samples were frozen in liquid nitrogen and stored at −80 °C until extraction.

### 4.2. RNA-seq

For the small size of *D. citri*, we concentrated the tissues of the insects by dissecting newly emerged adults (3 days old). A total of 2000 antennas (includes a modicum of tissues of heads), 200 heads (remove antennas), 150 thoraxes, 300 legs, 150 abdomens, and 1000 terminal abdomens (cut from the 5th abdominal segments) were collected from males, and the tissues from females had equal numbers. Total RNA of each sample was extracted using TRIzol Reagent (Invitrogen, Waltham, MA, USA). Total RNA of each sample was quantified and qualified by Agilent 2100 Bioanalyzer (Agilent Technologies, Palo Alto, CA, USA), NanoDrop (Thermo Fisher Scientific, Waltham, MA, USA). NEBNext^®^ Ultra™ RNA Library Prep Kit for Illumina^®^ (Illumina, San Diego, CA, USA) was used for next-generation sequencing library preparations. Then, we employed Agilent 2100 Bioanalyzer (Agilent Technologies, Palo Alto, CA, USA) for library quality evaluation. The libraries were sequenced on an Illumina HiSeq2500 the clean data was assembled by Trinity [62].

### 4.3. Identification of the Neuropeptides and Their Putative G-Protein-Coupled Receptors in D. citri

The amino acid sequences of *D. melanogaster*, *B. mori*, *N. lugens*, *C. suppressalis* and other arthropods were used as BLAST queries to search for the candidate neuropeptides and neuropeptides receptor genes from *D. citri* transcriptomic data. The E-value threshold for neuropeptides was 10, and the E-value threshold for receptors was 10^−5^. The candidate neuropeptides and neuropeptides receptor genes were reconfirmed by means of BLASTX analysis with the non-redundant protein sequence (NR) at NCBI (http://www.ncbi.nlm.nih.gov/).

### 4.4. Structure and Domain Analysis

The open reading frames (ORFs) of the candidate genes were predicted by the ORF finder (http://www.ncbi.nlm.nih.gov/). SignalP 4.1 Server (http://www.cbs.dtu.dk/services/SignalP/) was employed to identify neuropeptide signal peptide [63]. The transmembrane domains of putative neuropeptides receptors genes were notarized by DAS-TMfilter server (http://mendel.imp.ac.at/sat/DAS/) [64]. Pfam (http://pfam.xfam.org) [65] and Conserved Domains (https://www.ncbi.nlm.nih.gov/Structure/cdd/wrpsb.cgi?) [66] were used to search the domain.

### 4.5. Phylogenetic Analysis

Phylogenetic analysis of *D. citri* neuropeptides receptors was performed by comparing the neuropeptide GPCRs with those of *D. melanogaster*, *B. mori*, *N. lugens*, and other arthropods. For the *Drosophila*, the CG numbers of the sequences were used, and for the other species the original names of GPCRs in the publications were used, and the names of ligands were added if identified, the amino acid sequences can be seen in Supplementary Text S1. Sequences were aligned by ClustalW [67], and Neighbor-Joining trees were constructed in MEGA6 with 1000 bootstrap replicates [68]. The dendrograms were viewed in FigTree and edited in Adobe PhotoShop CS6.

### 4.6. Gene Expression Profiling of the Neuropeptides and Neuropeptide Receptors

Gene expression levels for each tissues sample of male and female antenna (included a modicum of tissues of heads), head (remove antenna), thorax, leg, abdomen, and abdomen terminal were estimated by RSEM (RNA-Seq by Expectation-Maximization) (v1.2.6) [69]. The expression levels were given as FPKM values (fragments per kilobase of transcript, per million fragments sequenced). Each of the FPKM values were transformed into log2 (RPKM + 1) values, and the expression profiling of the putative genes was generated and visualized by Heatmap Illustrator version 1.0 (http://hemi.biocuckoo.org/) [70]. Differential expression analysis used the DESeq package [71], six genes were selected to verify the accuracy of expression level by qRT-PCR (Supplementary Figure S2). The data analysis was conducted using GraphPad Prism 7.01. Statistical significance was evaluated using a one-way ANOVA followed by Tukey’s multiple range test at the 0.05 level.

### 4.7. Developmental Stages Expression Analysis

qRT-PCR was used with SYBR-green fluorescence. Total RNA was extracted from eggs, nymphs (1st to 5th-instars), and adults of one day and five days (sexual maturity) after eclosion. The PrimeScript™ RT reagent Kit with gDNA Eraser (Takara, Dalian, China) was used for cDNA synthesis following manufacturer’s instructions, reference gene *Dcitactin-1* was used as the internal controls [72]. The gene-specific primers were designed by Primer 3 program (http://elixir.ut.ee/Main/Services) [73] (Supplementary Table S2). The qRT-PCR reactions run on a CFX96 Touch™ Real-Time PCR System (Bio-Rad, California, USA), and the following program was adopted: 95 °C for 3 min, 40 cycles of 95 °C for 5 s and 59 °C for 30 s, and a final melting cycle (from 60 to 95 °C). Each experiment consisted of three biological replicates and three technical replicates. The relative values of mRNA expression were calculated by The 2^−ΔΔ*C*T^ method [74]. Data analysis was conducted using GraphPad Prism 7.01. Statistical significance was evaluated using a one-way ANOVA followed by the Tukey’s multiple range test at the 0.05 level.

## 5. Conclusions

The main purpose of this study was to identify the genes that encoding neuropeptides and their putative receptors. Transcriptomic analysis of these genes revealed the neuropeptide system in *D. citri*. In total, 40 neuropeptide and 42 neuropeptide receptor genes were identified. Most of these genes were annotated for the first time in *D. citri*. The expression of all these genes in different tissues was analyzed based on transcriptome profiling using RNA-seq data. The genes show high expression in the antenna and/or abdomen was speculated as representative potential pesticides or targets. Furthermore, these data contain more genes that are appropriate as new pesticides or targets which we did not mention; future research will prove that they are valuable in insect pest management.

## Figures and Tables

**Table 1:** Neuropeptides identified from *D. citri*. ORF, open reading frame; SP, signal peptide; aa, amino acid.

Neuropeptide Name	Accession No.	Acronym	ORF (aa)	SP (aa)	Homology Search with Known Protein		
					Species	Protein ID	E-Value
Adipokinetic hormone	MG550150	AKH	74	24	*Nilaparvata lugens*	AFN26934.1	6.00e-17
Allatostatin A	MG550151	AstA	181	21	*Rhodnius prolixus*	ACX47066.1	2.00e-21
Allatostatin B	MG550152	AstB	218	22	*Plautia stali*	BAU88428.1	2.00e-34
Allatostatin C	MG550153	AstC	98	23	*Pseudomyrmex gracilis*	XP_020296756.1	1.00e-20
Allatostatin CC	MG550154	AstCC	117	21	*Plautia stali*	BAV78791.1	6.00e-27
Allatotropin	MG550155	AT	152	28	*Nilaparvata lugens*	BAO00936.1	1.00e-08
Arginine vasopressin-like peptide	MG550156	AVLP	170	21	*Nilaparvata lugens*	BAO00934.1	1.00e-04
Bursicon beta subunit	MG550157	Burβ	134	19	*Nilaparvata lugens*	BAO00938.1	5.90e-52
Cardio acceleratory peptide 2b/Pyrokinin 1	MG550158	CAP2b	189	29	*Nilaparvata lugens*	BAO00941.1	1.00e-13
CCHamide 1	MG550159	CCH1	165	26	*Nilaparvata lugens*	BAO00942.1	8.00e-13
CCHamide 2	MG550160	CCH2	169	28	*Bombyx mori*	BAG55002.1	6.00e-08
CNMamide	MG550161	CNM	165	20	*Plautia stali*	BAV78799.1	9.00e-07
Corazonin	MG550162	Crz	155	26	*Solenopsis invicta*	XP_011172976.1	2.00e-03
Crustacean cardioactive peptide	MG550163	CCAP	149	25	*Plautia stali*	BAV78802.1	4.00e-42
Pyrokinin 2/Pheromone biosynthesis activating neruopeptide	MG550164	PBAN	200	29	*Bemisia tabaci*	XP_018895833.1	5.00e-20
Diuretic hormone 31	MG550165	DH31	119	29	*Nilaparvata lugens*	BAO00939.1	2.00e-35
Diuretic hormone 45/splicing variant of CRF-DH	MG550166	DH45	246	24	*Nilaparvata lugens*	BAO00945.1	6.00e-15
Eclosion hormone 1	MG550167	EH1	80	25	*Diuraphis noxia*	XP_015365040.1	1.00e-34
Eclosion hormone 2	MG550168	EH2	84	28	*Nilaparvata lugens*	BAO00951.1	2.00e-19
Ecdysis triggering hormone	MG550169	ETH	152	20	*Plautia stali*	BAV78804.1	2.00e-06
Elevenin-like peptide	MG550170	ELP	128	26	*Nilaparvata lugens*	BAO00952.1	3.00e-06
Glycoprotein hormone beta 5	MG550171	GPβ5	138	18	*Nilaparvata lugens*	BAO00956.1	3.00e-53
ITG-containing peptide	MG550172	ITG	216	21	*Agrilus planipennis*	XP_018325057.1	2.00e-81
Insulin-like peptide 1	MG550173	ILP1	136	25	*Nilaparvata lugens*	AIY24645.1	1.00e-03
Insulin-like peptide 2	MG550174	ILP2	147	20	*Nilaparvata lugens*	BAO00958.1	4.00e-04
Ion transport peptide like	MG550175	ITPL	124	26	*Harpegnathos saltator*	XP_011137372.1	2.00e-51
Kinin	MG550176	Kin	327	28	*Plautia stali*	BAV78814.1	6.00e-17
Myosuppressin	MG550177	MS	100	22	*Nilaparvata lugens*	BAO00963.1	6.00e-32
Natalisin	MG550178	NTL	214	25	*Chilo suppressalis*	ALM30330.1	8.70e-02
Neuroparsin	MG550179	NP	96	19	*Bombyx mori*	BAG50366.1	1.00e-13
Neuropeptide-like precursor 1	MG550180	NPLP1	617	19	*Nilaparvata lugens*	BAO00966.1	3.00e-05
neuropeptide-like precursor 3	MG550181	NPLP3	83	16	*Anoplophora glabripennis*	XP_018572912.1	7.00e-03
Orcokinin	MG550182	OK	164	17	*Nilaparvata lugens*	BAO00969.1	2.00e-30
Pigment dispersing factor	MG550183	PDF	82	25	*Nilaparvata lugens*	BAO00970.1	1.00e-07
Proctolin	MG550184	Pro	72	28	*Nilaparvata lugens*	BAO00972.1	1.20e-02
Prothoracicotropic hormone	MG550185	PTTH	168	31	*Nilaparvata lugens*	BAO00973.1	1.00e-09
RYamide	MG550186	RY	165	24	*Chilo suppressalis*	ALM30346.1	1.80e-02
Short neuropeptide F	MG550187	sNPF	110	27	*Nilaparvata lugens*	BAO00976.1	2.00e-17
SIFamide	MG550188	SIF	75	22	*Tribolium castaneum*	EFA07409.1	3.00e-17
Tachykinin	MG550189	TK	199	20	*Tribolium castaneum*	KYB25859.1	3.00e-14

**Table 2:** Neuropeptide G-protein-coupled receptor (GPCR) genes identified from *D. citri*. ORF, open reading frame; aa, amino acid; TMDs, transmembrane domains.

Receptor Name	Accession No.	ORF (aa)	TMD (No.)	Putative Identification	Species	Matched Gene (Accession No.)	E-Value
Neuropeptide receptor A1	MG550190	439	8	Allatostatin receptor type C	*Carausius morosus*	AOV81581.1	2e-179
Neuropeptide receptor A2	MG550191	392	7	Allatostatin-A receptor-like	*Nilaparvata lugens*	XP_022192722.1	6e-168
Neuropeptide receptor A3	MG550192	434	7	Neuropeptide GPCR A3	*Nilaparvata lugens*	BAO01052.1	3e-136
Neuropeptide receptor A4	MG550193	542	7	SIFamide receptor	*Onthophagus taurus*	XP_022919670.1	9e-162
Neuropeptide receptor A5	MG550194	381	7	QRFP-like peptide receptor	*Halyomorpha halys*	XP_014285975.1	1e-137
Neuropeptide receptor A6	MG550195	1185	6	Neuropeptide GPCR A6b	*Nilaparvata lugens*	BAO01056.1	7e-143
Neuropeptide receptor A7	MG550196	439	7	Neuropeptide receptor A7	*Nilaparvata lugens*	BAO01057.1	5e-163
Neuropeptide receptor A8	MG550197	520	7	G-protein coupled receptor moody	*Cimex lectularius*	XP_014257264.1	0
Neuropeptide receptor A9	MG550198	383	7	G-protein coupled receptor moody isoform X1	*Halyomorpha halys*	XP_014287792.1	2e-152
Neuropeptide receptor A10	MG550199	406	7	Sex peptide receptor	*Bemisia tabaci*	XP_018916635.1	0
Neuropeptide receptor A11	MG550200	410	7	Thyrotropin-releasing hormone receptor-like	*Diaphorina citri*	XP_008487532.2	0
Neuropeptide receptor A12	MG550201	438	7	Neuropeptide receptor	*Diaphorina citri*	AWT50635.1	0
Neuropeptide receptor A13	MG550202	411	7	Myosuppressin receptor	*Rhodnius prolixus*	AGT02812.1	8e-146
Neuropeptide receptor A14	MG550203	414	7	CCHamide-1 receptor	*Cimex lectularius*	XP_014250319.1	3e-173
Neuropeptide receptor A15	MG550204	378	7	CCHamide-2 receptor	*Melanaphis sacchari*	XP_025191408.1	2e-177
Neuropeptide receptor A16	MG550205	439	7	Neuropeptide GPCR A16	*Nilaparvata lugens*	BAO01066.1	5e-137
Neuropeptide receptor A17	MG550206	362	5	Neuropeptide GPCR A17	*Nilaparvata lugens*	BAO01067.1	7e-56
Neuropeptide receptor A18	MG550207	432	7	Neuropeptide GPCR A18	*Nilaparvata lugens*	BAO01068.1	3e-126
Neuropeptide receptor A19	MG550208	460	7	Neuropeptide GPCR A19	*Nilaparvata lugens*	BAO01069.1	1e-132
Neuropeptide receptor A20	MG550209	426	7	RYamide receptor	*Zootermopsis nevadensis*	XP_021924354.1	2e-125
Neuropeptide receptor A21	MG550210	405	7	Corazonin receptor	*Pyrrhocoris apterus*	ARV86500.1	2e-156
Neuropeptide receptor A22	MG550211	461	7	RYamide receptor-like	*Nilaparvata lugens*	XP_022187836.1	4e-153
Neuropeptide receptor A23	MG550212	448	7	Neuropeptide GPCR A23	*Nilaparvata lugens*	BAO01073.1	0
Neuropeptide receptor A24	MG550213	423	7	Tachykinin receptor	*Periplaneta americana*	ARK07245.1	0
Neuropeptide receptor A25	MG550214	463	7	Capa receptor-like	*Bemisia tabaci*	XP_018899674.1	8e-178
Neuropeptide receptor A26	MG550215	349	7	Neuropeptide GPCR A43	*Nilaparvata lugens*	BAO01093.1	6e-109
Neuropeptide receptor A27	MG550216	322	7	Cardio acceleratory peptide receptor	*Nilaparvata lugens*	XP_022190770.1	1e-167
Neuropeptide receptor A28	MG550217	543	7	Neuropeptide F receptor	*Rhodnius prolixus*	AKO62911.1	2e-48
Neuropeptide receptor A29	MG550218	383	7	Pyrokinin-1 receptor	*Rhodnius prolixus*	AFO73269.1	1e-154
Neuropeptide receptor A30	MG550219	552	7	Pyrokinin-1 receptor	*Cimex lectularius*	XP_014246737.1	8e-122
Neuropeptide receptor A31	MG550220	520	7	Pyrokinin-1 receptor	*Cephus cinctus*	XP_024938460.1	6e-113
Neuropeptide receptor A32	MG550221	493	6	AKH receptor	*Rhodnius prolixus*	AKO62857.1	1e-96
Neuropeptide receptor A33	MG550222	365	7	AKH receptor	*Pseudoregma bambucicola*	AKH80288.1	2e-151
Neuropeptide receptor A34	MG550223	419	7	Natalisin receptor	*Bactrocera dorsalis*	AQM36729.1	7e-144
Neuropeptide receptor A35	MG550224	423	7	Neuropeptide GPCR A41	*Nilaparvata lugens*	BAO01091.1	2e-123
Neuropeptide receptor B1	MG550228	408	7	Neuropeptide GPCR B1	*Nilaparvata lugens*	BAO01101.1	4e-164
Neuropeptide receptor B2	MG550229	463	7	PDF receptor	*Nilaparvata lugens*	XP_022204430.1	0
Neuropeptide receptor B3	MG550230	420	7	Neuropeptide receptor B4	*Chilo suppressalis*	ALM88344.1	4e-139
Neuropeptide receptor B4	MG550231	415	7	Diuretic hormone receptor	*Bemisia tabaci*	XP_018897695.1	0
Leucine-rich repeat G-protein-coupled receptor 1	MG550225	707	7	Lutropin-choriogonadotropic hormone receptor	*Drosophila rhopaloa*	XP_016976568.1	0
Leucine-rich repeat G-protein-coupled receptor 2	MG550226	817	7	Lutropin-choriogonadotropic hormone receptor-like	*Cryptotermes secundus*	XP_023716984.1	0
Leucine-rich repeat G-protein-coupled receptor 3	MG550226	1011	7	Neuropeptide GPCR A47	*Nilaparvata lugens*	BAO01097.1	0

## Supplementary Materials

Supplementary materials can be found at http://www.mdpi.com/1422-0067/19/12/3912/s1.
