# Supplementary material for: Characterization and Expression Profiling of Neuropeptides and G-Protein-Coupled Receptors (GPCRs) for Neuropeptides in the Asian Citrus Psyllid, Diaphorina citri (Hemiptera: Psyllidae)
Source: Int J Mol Sci. 2018 Dec 6;19(12):3912. doi: 10.3390/ijms19123912 (PMC6321106; doi:10.3390/ijms19123912)
Supplement: Supplementary file 1 [file ijms-19-03912-s001.zip › Figure S2 The gene expression level of neuropeptides and neuropeptide receptors in tissues of the adult citrus psyllid.pdf]

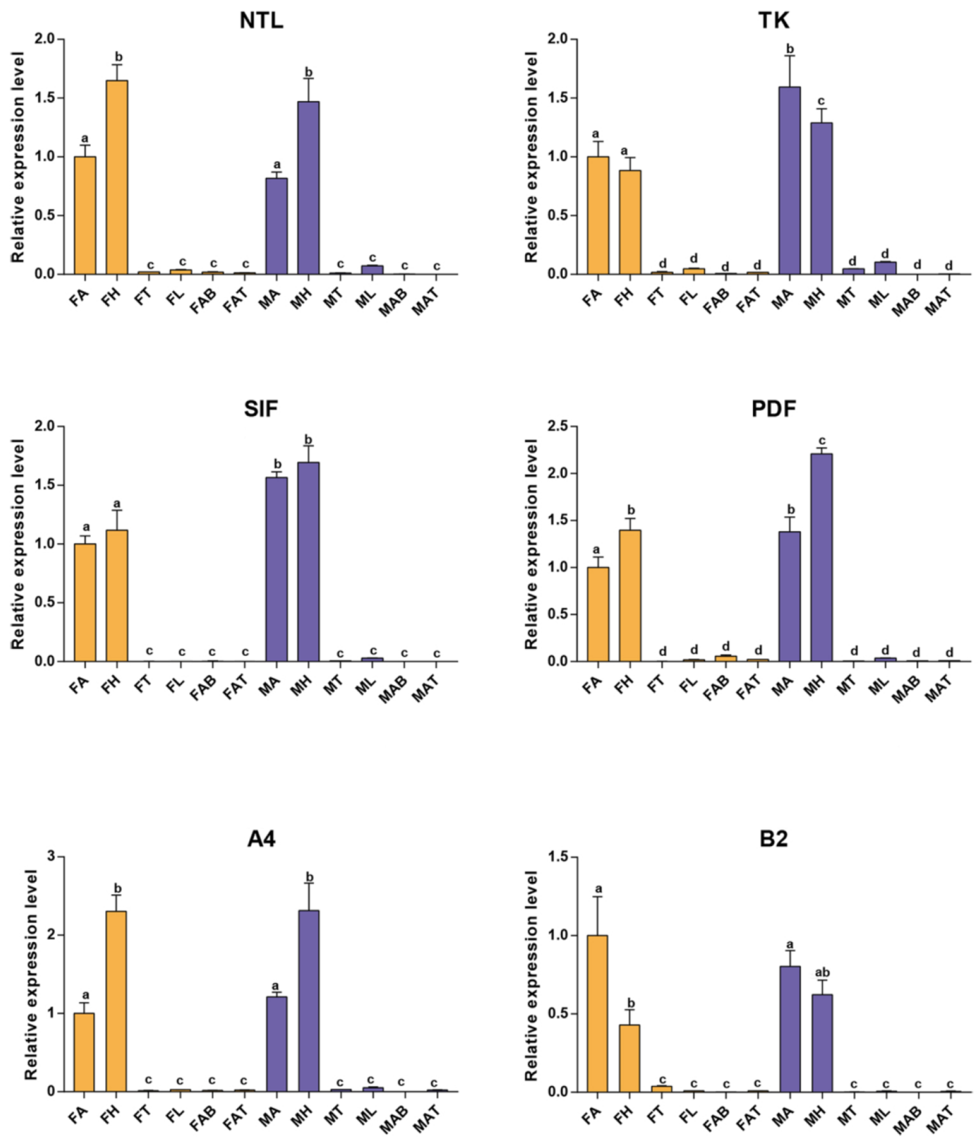

Figure S2. The gene expression level of neuropeptides and neuropeptide receptors in tissues of the adult citrus psyllid. The Y-axis represents the relative expression level and the X-axis the tissue type. Standard error is represented by the error bar and signifcant differences are represented by the different letters ($p < 0.05$). FA, female antenna; FH, female head; FT, female thorax; FL, female leg; FAB, female abdomen; FAT, female abdomen terminal; MA, male antenna; MH, male head; MT, male thorax; ML, male leg; MAB, male abdomen; MAT, male abdomen terminal.
