# Supplementary material for: Characterization and Expression Profiling of Neuropeptides and G-Protein-Coupled Receptors (GPCRs) for Neuropeptides in the Asian Citrus Psyllid, Diaphorina citri (Hemiptera: Psyllidae)
Source: Int J Mol Sci. 2018 Dec 6;19(12):3912. doi: 10.3390/ijms19123912 (PMC6321106; doi:10.3390/ijms19123912)
Supplement: Supplementary file 1 [file ijms-19-03912-s001.zip › Table S1 Neuropeptide genes in Diaphorina citri and other insects.docx]

Table S1 Neuropeptide genes in *Diaphorina citri* and other insects. +, identified; -, absence or no find.

| Peptide | *Diaphorina*  *citri* | *Acyrthosiphon*  *pisum* | *Nilaparvata*  *lugens* | *Drosophila*  *melanogaster* | *Bombyx mori* | *Apis mellifera* | *Tribolium*  *castaneum* |
| --- | --- | --- | --- | --- | --- | --- | --- |
| AKH | + | + | + | + | + | + | + |
| ACP | - | - | + | - | + | - | + |
| AstA | + | + | + | + | + | + | - |
| AstB | + | + | + | + | + | + | + |
| AstC | + | + | + | + | + | + | + |
| AstCC | + | + | + | + | + | + | - |
| AT | + | + | + | - | + | + | + |
| AVLP | + | - | + | - | - | - | + |
| Burα | - | + | + | + | + | + | + |
| Burβ | + | + | + | + | + | + | + |
| CAPA | + | + | + | + | + | + | + |
| CCH 1 | + | + | + | + | + | + | + |
| CCH 2 | + | + | + | + | + | + | + |
| CNM | + | - | + | + | - | + | + |
| Crz | + | - | + | + | + | + | - |
| CCAP | + | + | + | + | + | + | + |
| PBAN | + | + | + | + | + | + | + |
| DH31 | + | + | + | + | + | + | + |
| DH34 | - |  | - | - | + | - | + |
| DH41 | - | - | + | + | + | + | + |
| DH45 | + | + | + | - | + | - | - |
| ETH | + | + | + | + | + | + | + |
| EH | + | + | + | + | + | + | + |
| ELP | + | + | + | - | + | + | + |
| FMRF | - | + | + | + | + | + | + |
| GPA2 | - | + | + | + | + | - | + |
| GPB5 | + | + | + | + | + | - | + |
| IMF | - | - | - | - | + | - | - |
| ILP | + | + | + | + | + | + | + |
| ITG | + | - | + | + | + | + | + |
| ITP | - | + | + | + | + | + | + |
| ITPL | + | + | + | + | + | + | + |
| Kinin | + | + | + | + | + | + | - |
| MS | + | + | + | + | + | + | + |
| NTL | + | + | + | + | + | + | + |
| NP | + | - | + | - | + | + | + |
| NPF | - | + | + | + | + | + | - |
| NPLP | + | + | + | + | + | + | + |
| OK | + | + | + | + | + | + | + |
| PDF | + | - | + | + | + | + | - |
| Pro | + | + | + | + | - | - | + |
| PTTH | + | - | + | + | + | - | + |
| RY | + | + | + | + | + | + | + |
| sNPF | + | + | + | + | + | + | + |
| SIF | + | + | + | + | + | + | + |
| SK | - | - | + | + | + | + | + |
| TK | + | + | + | + | + | + | + |
| TR | - | - | - | + | + | - | + |
