# Supplementary material for: Characterization and Expression Profiling of Neuropeptides and G-Protein-Coupled Receptors (GPCRs) for Neuropeptides in the Asian Citrus Psyllid, Diaphorina citri (Hemiptera: Psyllidae)
Source: Int J Mol Sci. 2018 Dec 6;19(12):3912. doi: 10.3390/ijms19123912 (PMC6321106; doi:10.3390/ijms19123912)
Supplement: Supplementary file 1 [file ijms-19-03912-s001.zip › Table S2 Sequences for quantitative real-time PCR primers..docx]

Table S2 Sequences for quantitative real-time PCR (qRT-PCR) primers.

| Gene | Forward Primer sequence (5’ to 3’) | Reverse Primer sequence (5’ to 3’) |
| --- | --- | --- |
| AKH | AGTCATGTTCCTAGTCGTGTTGT | TGGAGCTGCGACATTCATCA |
| AstA | CAGTTACATCATGGCGGACC | ATATTGCTTGCCACGCTTGT |
| AstB | GAAGAGAAGAGGGCGTGGAA | AGGTTATCGTCGGGCTGAAA |
| AstC | CGACAGCTTTCAGACAAACGA | AATAACTTCGCTTCCGCTGC |
| AstCC | CAAAACGAGCGGCCTTACTT | CAGTACGTGCGACCTTTCTG |
| AT | CATCGACGGCAGGAGGATAT | GGGGTTGGTTTGAAGTTGCT |
| AVLP | GACCCAGGCTCAAAATGTGG | CCCACGCTTTTATCTCGACAC |
| Burβ | CGGTCAACAAATGTGAGGGG | GCGTTGCCATCTTCTCACTG |
| CAP2b | AGGAAGAGGCAGTGATGACC | CGCGGACCAAACCACATATT |
| CCH1 | TCTTCGGCCCTCAGACATTT | CCAGTTTTCGGGCTTGACTC |
| CCH2 | CTGCCAGCGACGACTATTTC | GTGTCAGGCGGGCTCATA |
| CNM | TCAGTACGAGAGCAGCAACA | TCATTTCGTAGCCAGGGTGT |
| Crz | CCTCGTCATCTCCTTGCCAT | GACTGACTTTTCCCCTCCCC |
| CCAP | GCGTTACATGGTGTTGGTCC | AGTTCCCATTGACTCCGAGG |
| PK | CCCTGACAACCCTCTACTGG | CTCCTCTCCCTCTTCCTCCT |
| DH31 | TGTAGAGGGTGCGGATCAAC | TGTTTGGCTGCTTGTGATCC |
| DH45 | TGAAGATGCCTGGGACGATT | GCCGTTGTTCTTCCAGTTGG |
| EH1 | TCTTCTCCTCTCCGCCCTAG | CAGTCCGGGATCATTTTGCC |
| EH2 | GTGGTCGGGTACTTTGGGG | GCGTAGAGTGTTCGGGTTGT |
| ETH | GGGTCCTCGTGTTTCTCTCA | GGACTGACTTGGCTGCTTTG |
| ELP | ATGTTGTTCGTGCTAGAGATTCG | AACCTCGTTGTCTTCGTCATTTT |
| GPβ5 | TACAAGCGATCCCACCATCC | CGCCTCAGACGACTTACACA |
| ITG | GAGAGCTATGCCGTCGAGAT | GCCTGTCTATGTCTGCGTTG |
| ILP1 | CACTCATCGGTCTGCTCCTC | ACTGGGTACTTCGTTTGCCA |
| ILP2 | TACACCACCAATGAACCCGA | GGGTGAACAATGTGCAAGCT |
| ITPL | CTCAGCAAGCGGTCGTTTT | ATCCGCCTGTATTGAATCCGA |
| Kin | CTGATCCCCTACAAGCAGCT | TTCTCTGCCAAGTCCATGCT |
| MS | TATGCCACCTCTCCAATGCA | GCTGATTGATCTCTGGGTTCAC |
| NTL | GTCCTTCCGAGCGACTGTAT | CGTGTTCCGTTTCCAGATCG |
| NP | CAGCTACTGTCTAGGCCGG | CCACAGGGTTGATTTTCCGC |
| NPLP1 | CCTCCAGTGTACTACCAGCC | GACTGCGTTTGTCTTGACCAT |
| NPLP3 | CCTGCCCCTGAACCCAAA | CAGGCACGGAATGGACAAC |
| OK | CGAGACTTGGACGCTCTGA | TTTCATCCATGCCATTCCGC |
| PDF | TCCTGTTCATATTGGGTGCTCA | GGACCTCTGCTGTACCAACT |
| Pro | GCTGCTATTGAGTTGCTTCTGTA | TCTGAGTAGTTCCTTGAGTTTGC |
| PTTH | CATCTTTCAAAGTCCGGGCG | CGTAGAACCCATGCCAACAC |
| RY | AGACTTCTATCTTGGCACTCGT | TGGGAACTGGTAGATTACTCCG |
| sNPF | CCTCAGCAAGCGGTAGATCT | TATCAGTGAGGCCAGGTTCC |
| SIF | GTTGTGTTCATCGTCTCCACC | TCTCACACATGGATGCAAAAGTT |
| TK | CTACGAATCCAAACGAGCGC | CCCGCATACCAAAGAAGCTC |
| A1 | TATGTCCTCTGCTGGCTTCC | TGCACGCCTTGAGAAAACTC |
| A2 | CATCGTGTGTTGTGTTCCGT | CGACATGGACGCTATGGGAT |
| A3 | GTTACATCTGAGCCCGCTTG | TCTCTTTGATGCCTGCCTCA |
| A4 | CTGTGGAGATGGGTGGGAAT | CGACAGCAATGACCAAGGAG |
| A5 | TCCTCCTGTGGCGAATCTAC | GTCTTGTGGCAGAGTCGTTG |
| A6 | CAGTTTCCGGCCTACATTCG | CAGCAACACCATCAGATCCG |
| A7 | TGGCTCAACGACAATTTCCG | TCATCGTCTATCTTGCCCGG |
| A8 | ACTGATGACTCCGCCATAGG | GCCAGAGGAGAGAGGAAGTG |
| A9 | CGTTTTGACCTGGACACGAG | CTGGATTTTCGAGGTGTGCC |
| A10 | GTGGTCACGGTCTTCCTTCT | GGGGTAGCTGACGATGATGA |
| A11 | TAGGATCTGCACGCTGAACA | CACGAGATAGTAGGAGCGGG |
| A12 | CCACGCCTGTACTGTTCCTA | ACGGGGAGTAGTGTAGGTCA |
| A13 | CTGACGATTGTGTTGCGGAT | CTGACGATTGTGTTGCGGAT |
| A14 | GTACACGTTTGAGTCCTGGC | CTAGTAGCTCGCTTCCCTCC |
| A15 | GTTCCCTTCACTTCCACCCT | CAGAGGGTAGAGCGAGGATG |
| A16 | AACGTCCCGAGCTAAGAACA | AAGCAATCGACATCAACCCG |
| A17 | CATCATTCCATGCCTGACCG | CTCGTTCTTCCTCAATGCGG |
| A18 | GTATGGTTTGTGGTGGCGTT | TGCTGTTTTGTCTGGTGTCG |
| A19 | ATTTTCGGGCGGAGATTTCG | TACGGATGGTGGGTATGGTG |
| A20 | TAGTGGTGTGGGGTAAGCAG | CGGAAACCATTTGAGCTGCT |
| A21 | TCTACGGAGCTTTCCACCTG | GTAGCCGTTCCCATTTCGAC |
| A22 | ATCCTAACCACGAACAGGCA | CAAACTCAACACAGCCGTCA |
| A23 | GTACAGGCGAGTTTTAGCGG | GTGCCACAACTATGACCAGC |
| A24 | CTATCTGTGCGAGCGTGTTC | CACTGTCCGAACTTTGCCAT |
| A25 | CCCTCCTCTCCTATGCCATG | AGAATCCGCTCTCGTACAGG |
| A26 | TATGCGGGTTTGTGCTCCTA | GCTCTCCAAGACCTCCTGTT |
| A27 | ATGGCACTGTACTCCCAACA | GACACAAAGATCTGCCACCG |
| A28 | ACTCCCTCGTCTCGTTCATC | AATAGTTCCGCTGGTCACCA |
| A29 | TGCCATCTGTCATCCGTTCT | ACAATGTGCTTCCTCCTCGT |
| A30 | CACCTACTCCGCTCTCATCC | CGGGTCACATAGGGAGTCTC |
| A31 | CTACTACCTGTCTCCTGCGG | CCGATGTTCCCCGAGTGATA |
| A32 | GTCTGTATTTGTCGTCGGGC | CGAGGCGAAAGAGTTGAAGG |
| A33 | TCATTCCCGTCACGTGGTTA | CCTGGACTTTGCCCTTCCTA |
| A34 | AGACTACATGCTGCGGAACA | GACAACACTCCAAGCCAGC |
| A35 | CTCTACTCTCGCCGCCATTA | TTTCACCAGCTTGCATGTCC |
| B1 | CAGGTTCCAGAGCGTTTGTC | TTCACCTTTTGCCGCATCTC |
| B2 | CGCTTCAGGAGTCTCAGGAA | AACAGGACGTAGGAGGCTTC |
| B3 | CCAAACTCCACAGCAACTCC | TGACTAGACACGAGGACAGC |
| B4 | ATGTTAGCCTGTACGCCCAT | GACCAGTCTCGGCTACAAGT |
| LGR1 | TCCCGTATCCAATGACCTCG | TCAGCTCTTGATTCCCACGT |
| LGR2 | ACGCATGGCTCTGTTAGTCT | CGTACAAGTAAGGGTTCGCG |
| LGR3 | GGCGATCATCTTCCCGTTTC | AAACAAACGCCCGATCTTCC |
