# Supplementary material for: Characterization and Expression Profiling of Neuropeptides and G-Protein-Coupled Receptors (GPCRs) for Neuropeptides in the Asian Citrus Psyllid, Diaphorina citri (Hemiptera: Psyllidae)
Source: Int J Mol Sci. 2018 Dec 6;19(12):3912. doi: 10.3390/ijms19123912 (PMC6321106; doi:10.3390/ijms19123912)
Supplement: Supplementary file 1 [file ijms-19-03912-s001.zip › Figure S1 The DcTK and DcNTL peptides show typical C-terminal motifs.pdf]

```
>DcTK
MNFLWLGCLWLATVISVTSSQDTDKRAPKPNAFA
AMRGKKDFDYDSGFYYDKRVPMGFAGVRGKKLSD
DYSSFYESKRAPSRSFFAVKGKRELLTPDLSSLL
DDIQVPDMDKRAPARFFGMRGKKGPSSQSFFGMR
GKKDYDLEPYWYSRAGYSDDLSQLLNVLNAPDTP
TGRAKRDVSDMLTANSLGLSSPSQHSADN
```

(**a**)

```
DcTK1   QDTDKRAPKPNAFAAMRa
DcTK2   ---------VPMGFAGVRa
NlTK1   -------GSNMGFVGMRa
NlTK2   --------GSMGFVGMRa
NlTK3   ---------AVGFVGMRa
NlTK4   ---------AMGFMGMRa
NlTK5   ---------AMGFQGMRa
DcTK3   --------APARFFGMRa
DcTK4   ------GPSSQSFFGMRa
```

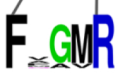

(**b**)

```
>DcNTL
MSLVLGYYVVSILVHFLNQINGAHSQERIIRSSIRSMLENQLSPDFYPTRGRRAKS
SEEEVDEPPFWAQRGRSLDVLSRTWPRNVELSSSNSLEDLPNFWASSGHQSPGNDK
EIPFWANRGRSVLPSDCIDPNYSLAYAEEPRVVVIERRDNSDDDHNYSNFWASRGR
RDQYGLMKHRLNKAIWKRNTIKLTRGNEKPSKYDERKPLSGDNGDH
```

(**c**)

```
DcNTL1   -------------------------QERIIRSSIRSMLENQLSPDFYPTRa
NlNTL1   -----------------------ENGHRVSRSDVRAVLGEKMDPNFWPTRa
NlNTL7   ----------------------APLATRQ--GENAARALGLSSDQFWPARa
NlNTL6   ---------------------------------GLLDTLGTAPNDFWASRa
DcNTL4   ----------------------------------DNSDDDHNYSNFWASRa
NlNTL5   ---------------------------------DDETAGGSSLVDPFWVARa
DcNTL3   SLDVLSRTWPRNVELSSSNSLEDLPNFW-ASSGHQSPGNDKEIPFWANRa
DcNTL2   ---------------------------------AKSSEEEVDEPPFWAQRa
NlNTL4   ---------------------SVRLGELQ-SLLQERGVGSDEEPPFWANRa
NlNTL2   ------------------------------SPLGAALGSEEDDAPFWANRa
NlNTL3   ----------------------------------SVRLGEEDEAPFWANRa
```

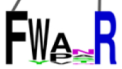

(**d**)

Figure S1. The DcTK and DcNTL peptides show typical C-terminal motifs. (a) and (c) Deduced amino acid sequences for the predicted precursors of the TK and NTL peptides from *D. citri*; underlined sequences, signal peptide; sequences in bold and blue/green colour, predicted TK/NTL-peptides; sequences in bold and italic, predicted amidation signal with dibasic cleavage sites. (b) and (d) Alignment of putative mature peptides of TK and NTL showing the C-terminal motifs with the variations; the sequences were aligned using Clustal Omega [1], and the sequence logos for the C-terminal motifs were generated by WEBLOGO [2].

References

1. Sievers, F.; Higgins, D. G. Clustal Omega. *Curr Protoc Bioinformatics* **2014**, *48*, 3-13, doi:10.1002/0471250953.bi0313s48
2. Crooks, G. E.; Hon, G.; Chandonia, J. M.; Brenner, S. E. WebLogo: a sequence logo generator. *Genome Res.* **2004**, *14*, 1188-1190, doi:10.1101/gr.849004
